# Detecting hidden threats: proteomic changes in urinary extracellular vesicles are associated with pediatric kidney allograft rejection

**DOI:** 10.1038/s41390-026-04866-z

**Published:** 2026-04-22

**Authors:** Levy Erez Daniella, Eisner Sigal, Issa Erin, Sabah Idan, Levin Liron, Chalifa-Caspi Vered, Langman Shay, Landau Daniel, Haskin Orly, Alfandary Hadas, Tur-Kaspa Ran, Nesher Eviatar, Zemel Romy

**Affiliations:** 1 Schneider Children’s Medical Center of Israel, Petah Tikva, Israel; 2 Gray Faculty of Health Sciences,Tel Aviv University, Tel Aviv-Yafo, Israel; 3 Children’s Hospital of Philadelphia, Philadelphia, PA USA; 4 Molecular Hepatology & Transplantation Immunology Research Lab. Felsenstein Medical Research Center, Beilinson campus, Rabin Medical Center, Petah Tikva, Israel; 5 Department of Organ Transplantation, Rabin Medical Center, Petah Tikva, Israel; 6 Ilse Katz Institute for Nanoscale Science & Technology, Ben-Gurion University of the Negev, Beer-Sheva, Israel; 7 Liver Institute, Beilinson Campus, Rabin Medical Center, Petah Tikva, Israel; 8 Azrieli faculty of Medicine, Bar-Ilan University, Safed, Israel

## Abstract

**Background:**

Early detection of kidney allograft injury is essential to preserve long-term graft function. Current diagnostics, particularly biopsy, are invasive and may be inconclusive. Extracellular-vesicles (EVs) carry molecular cargo that reflects renal cellular states. Although EV proteomics has shown promise for detecting acute rejection (AR) in adults, its value in pediatrics(PKT) is not well defined.

**Methods:**

In this pilot study, urine samples from 29 PKT recipients and 3 healthy controls were analyzed, including 7 with biopsy-confirmed AR, 9 with chronic rejection (CR), and 13 with stable graft function (ST). Classification was based on urine findings, creatinine trends, and biopsy results. EVs were isolated from fresh-second-void urine by ultracentrifugation and analyzed using LC/MS. Longitudinal samples from three AR patients assessed temporal proteomic changes.

**Results:**

Overall, 192 proteins were differentially abundant across groups (FDR *p* < 0.05). AR showed enrichment of immune-related pathways and increased free hemoglobin proteins compared with ST, while CR closely resembled ST. EV concentration and size distribution were similar across groups, indicating proteomic differences were independent of EV abundance. Longitudinal analyses revealed dynamic proteomic changes corresponding to rejection and recovery.

**Conclusions:**

EV proteomics is a promising approach for detecting and monitoring AR in PKT recipients and warrants larger multicenter studies.

**Impact:**

- This pilot study demonstrates urinary extracellular vesicle (EV) proteomics as a promising non-invasive approach to detect and monitor acute rejection (AR) in pediatric kidney-transplant (PKT) recipients. EVs carry molecular cargo reflecting kidney cell states, providing a dynamic readout of graft health and injury. Early identification of rejection may reduce reliance on invasive biopsies, enable timely intervention, and support precision-based, personalized management. As the first study to characterize EV proteomic profiles in PKT, it lays important groundwork. Larger multicenter studies are warranted to validate these findings.

## Introduction

Kidney transplantation offers children with end-stage kidney disease the best chance of leading near-normal lives, markedly reducing both mortality and the day-to-day burden of dialysis.^1^ Although one-year graft survival now routinely surpasses 94%,^2,3^ maintaining kidney function through adolescence and beyond remains challenging. Graft survival curves for recipients 11–18 years old flatten and then fall during the teenage years, a period in which heightened alloimmunity, evolving pharmacokinetics, and lapses in adherence converge.^3^

AR  is one of the major forces driving graft loss. U.S. registry data shows biopsy-proven AR in as many as 11% of pediatric recipients within the first post-transplant year^4^. Outcomes depend on a host of variables; donor quality, HLA mismatch, recipient sensitization, and others,^5^ yet subclinical rejection (SCR) is emerging as an equally important concern. Histologic studies report SCR in 15–50% of apparently stable children, and these silent episodes accelerate chronic allograft damage.^6–9^

Surveillance today relies on serum creatinine and protocol or indication biopsies. Creatinine lacks sensitivity; biopsy, while definitive, is invasive and carries bleeding and anesthesia risks, issues magnified in younger, smaller patients. The field, therefore, needs reliable, non-invasive biomarkers that signal allograft stress early enough to permit pre-emptive therapy.

EVs have become attractive candidates. Released by virtually every cell, these membrane-enclosed nanoparticles ferry proteins, lipids, and nucleic acids that mirror the physiological state of their parent cells.^10–12^ Because kidney cells continuously shed EVs into urine, the urinary EV compartment functions as a real-time, organ-specific “liquid biopsy.” Changes in EV cargo track various forms of kidney injury.^13^ Adult transplant studies have identified EV signatures linked to rejection and other complications,^14–21^ but pediatric data are almost entirely lacking. Given developmental differences in immune regulation and kidney maturation, adult biomarkers cannot simply be extrapolated to children.

Here, we present the first pilot study of urinary EVs from PKT. Our goals were to (i) catalogue EV protein profiles across ST function (no major abnormalities), AR, and CR; (ii) isolate protein signatures that distinguish AR from stability; and (iii) map temporal shifts in the EV proteome before, during, and after rejection. Using high-resolution LC-MS and rigorous bioinformatics, we provide a foundation for EV-based, non-invasive monitoring tailored to the pediatric population.

While larger, multicenter studies are still needed, these data lay crucial groundwork. An accurate urinary EV biomarker panel could reduce biopsy frequency, guide immunosuppression more precisely, and ultimately lengthen the lives of pediatric grafts.

## Methods

### Patient and sample information

The study was a single-center pilot study of children followed at the Schneider Children’s Medical Center in Israel. The study was approved by the institutional review board at Rabin Medical Center, and parents and patients provided written informed consent following the Declaration of Helsinki. All patients undergoing a kidney biopsy between May 2021-January 2023 were approached as well as clinical stable kidney recipients with similar ages with no abnormalities (i.e.: without any cause for biopsy and with proper stable serum creatinine levels and estimated Glomerular filtration rate (eGFR) 3 months prior to enrollment and no evidence of hematuria or proteinuria). We enrolled 29 kidney-transplant recipients and 3 matched healthy controls. Among the 29 transplant recipients, 15 patients had an indication for a kidney biopsy at the time of sample collection.

Demographic and clinical characteristics, and information on the donors were recorded. Study data was collected and managed using a REDCap electronic data capture tool hosted at SCMCI.^22,23^ EGFR was calculated using the CKID equation.^24^ We used the center’s pathologist’s kidney transplant biopsy specimen report to define AR by the 2019 Banff Working Groups’ criteria.^6^ For our analysis, we combined acute cellular rejection (ACR) and mixed ACR+ antibody-mediated rejection (ABMR) to form the AR group. All patients in the study with rejection had TCMR(T cell mediated rejection) Grade 1 A and above. All patients with CR were defined as non-active ABMR, non-active TCMR with evidence of fibrosis. Given only one patient developed isolated ABMR he was excluded from the analysis. CR was defined as stable serum creatinine with a previous kidney biopsy compatible with CR.

A total of 35 second void urine samples (26 patients with a single sample, 3 patients with repeated samples, and 3 healthy controls with a single sample) were collected from patients with matching serum samples. Urine samples from patients undergoing a kidney biopsy were collected prior to biopsy to avoid hematuria contaminating the samples. all urine and serum samples were frozen at -80 ^o^C prior to collective analysis.

### Urine EV isolation and LC-MS analysis

In order to remove uromodulin, we centrifuged the urine at 3000 G for 20 minutes to remove debris and then the supernatant was centrifuged at 10,000 g at 4^o^C for 30 minutes and was filtered through a 0.22-micron filter. Ultracentrifugation was performed twice at 100,000 g for 70 min each cycle. The pellet containing EVs was analyzed on the Zetaview machine for Nano particle sizing and quantification (NTA). EV extraction, quantification and analysis adhered to MISEV2018 guidelines. Aliquots of 100 microliters were stored at -80. Proteomic analysis was performed at the Smoler Proteomics Center, Technion, Haifa. A total of 10^13^ particles were analyzed per patient for proteomics. Isolated EVs were first lysed in Tris buffer containing 5% SDS, followed by heating and sonication to ensure complete protein solubilization. Protein concentration was determined using the Bicinchoninic Acid Protein Assay(BCA assay) prior to enzymatic digestion. Proteins were then reduced, alkylated, and digested with trypsin according to standard protocols. The resulting peptides were desalted and analyzed by LC-MS/MS using a Q-Exactive HF mass spectrometer (Thermo Fisher Scientific).

Raw MS data was processed using MaxQuant v2.1.1.0 for protein identification and label-free quantification (LFQ). Protein identification was performed against the human UniProt database with a 1% FDR at both the peptide and protein levels. LFQ normalization was applied to account for variations in sample loading and instrument response. Additional data quality control steps included checking for peptide length distribution, missed cleavages, as well as EV marker enrichment including CD63, CD9, CD82.

### Bioinformatics and statistical analysis

In-depth bioinformatics analysis of the proteomics data was performed by the Bioinformatics Core Facility at Ben Gurion University. Variance stabilized expression (VSD) normalization, imputation and statistical analysis were done using the “DEP” R package. Missing values were imputed using the “MinProb” method. The statistical, clustering and enrichment analyses were applied using “all patients”, such that from patients with repeated samples only one sample was chosen (13B, 21 A, 35 A). The statistical tests considered batch and sex as confounders. For visual presentation, the batch effect was corrected using the “sva” R package. Differentially expressed proteins that were found to be significantly different between AR and ST (limma adjusted *p* value < 0.05) were submitted to hierarchical clustering (method: ward. D2, metric: Pearson). Identification of clusters was done using the “eclust” R package. Clustering was performed at the average gene expression level per patient group. The number of clusters was estimated using the “gap” statistic with the maximum number of allowed clusters set to 5. Subsequently, the proteins in each cluster were submitted to hierarchical clustering at the sample level, and all clusters were displayed as a heatmap. Up- and down-regulated proteins between AR and ST were further tested for enrichment of gene ontology (GO) biological process terms using the “clusterProfiler” R package. The background gene set was defined as the set of identified proteins. For presentation as a bar plot, the clusterProfiler “simplify” function was used to remove redundancy of enriched GO terms.

### Statistical analysis

Clinical Data was summarized as mean and standard deviation (SD) or median and interquartile range (IQR), as appropriate based on their distribution(using the Kolmogorov-Smirnov test) and compared using the Student *t*, ANOVA, and Kruskal–Wallis tests as appropriate. Categorical variables are presented as frequencies and percentages and compared using chi-square or Fisher’s exact tests, as appropriate. A P value < 0.01 was considered significant. Analyses were performed using Stata statistical software package 14.1 (TX).

## Results

### Cohort characteristics

Thirty-two children were recruited: 29 kidney-transplant recipients and three healthy controls (overall median age14.5years, IQR 13–19). Among the recipients, seven (24%) had biopsy-confirmed AR, nine (31%) had CR, and 13 (45%) had ST graft function. Graft vintage differed significantly between rejection phenotypes: AR occurred a median of 3 years after transplantation, whereas CR was detected a median of 10 years post-graft (*p* = 0.009).

All recipients received induction immunosuppression, interleukin-2-receptor blockade in 23 patients (80%), and anti-thymocyte globulin in six (20%). Maintenance therapy consisted of tacrolimus in 27 (95%), mycophenolate mofetil in all recipients, and low-dose corticosteroids in all recipients. Detailed demographic, immunosuppression regimens and clinical data are provided in Table 1.

**Table 1:** Demographic and clinical data of patients

Characteristic	Acute rejection (AR)	Chronic Rejection (CR)	Stable= no major abnormalities (ST)	*P* value
Number of patients	7	9	13	
Recipient age, years, median (IQR)	14.5 (13,19)	14 (8,18)	13 (10.5,17.5)	0.7
Gender, male% (n)	85% (6)	55% (5)	53% (7)	0.87
Time since transplant, years, median (IQR)	3 (0–5)	10 (7-15)	3 (2–6)	0.006
Cause of renal failure:				
CAKUT	42% (3)	14.2% (1)	35% (5)	0.9
Glomerular disease	0% (0)	28.4% (2)	23% (3)	
Genetic Causes	42% (3)	44% (4)	29% (4)	
Unknown	14% (1)	0% (0)	7% (1)	
Cadaveric donor	28%	29%	38%	0.8
Induction regimen:				
IL2RB	100% (7)	67% (6)	85% (11)	0.28
Thymoglobulin	0%	33% (3)	8.3% ((1	
Combined Rejection, % (n)	28% (2)	NA	NA	NA
Acute Cellular Rejection, % (n)	72% (5)	NA	NA	NA
Serum creatinine (mg/dl), median (IQR)	1.7(1.1–6.8)	1.49(1.3–2.4)	0.89 (0.6–1.12)	0.005
Donor Specific Antibodies positivity (%)	28% (2)	33% (3)	30% (4)	0.89
Urine Protein/Creatinine ratio, mg/mg (IQR)	1.2 (0.36–19)	0.1 (0.1–0.55)	0.12 (0.075–0.17)	0.01

Data are presented as median (IQR = interquartile range) or *n* (%). CAKUT, congenital anomalies of kidney and urinary tract; IL2RB- Interleukin-2 receptor blocker A *P* < 0.05 was considered significant.

The control group was comprised of three children aged 3, 9, and 12 years, two of whom were female; all demonstrated normal renal function.

### Extracellular-vesicle characterization

**Fig. 1:**
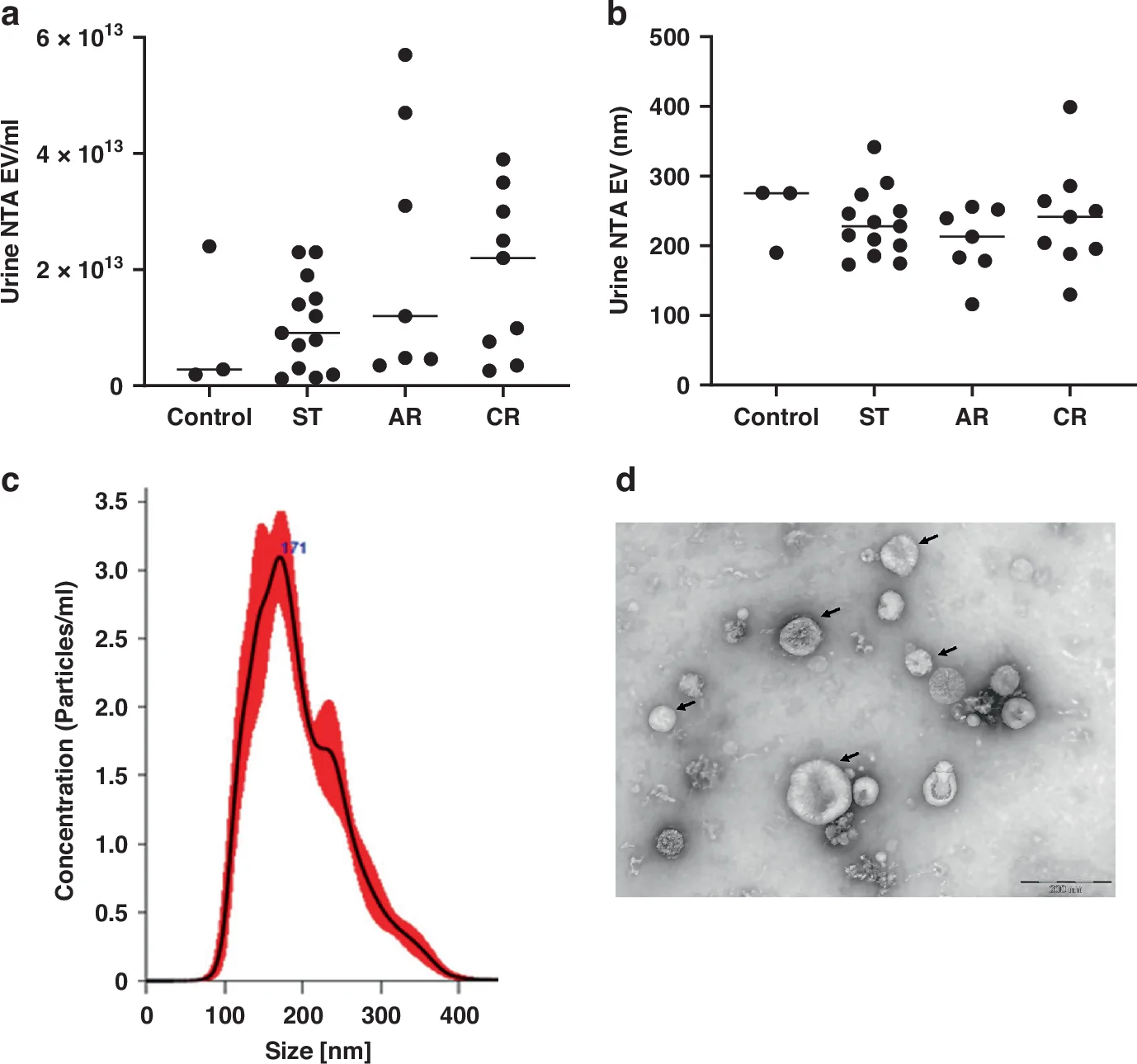
Characterization of urine-derived EV, size and number. **a** Urine EV concentration from the urine of kidney transplant patients. assessed by nanoparticle tracking analysis (NTA) after isolation by ultracentrifugation. (Healthy controls EV *n* = 3, ST n = 13, AR n = 7, and CR *n* = 9). Kruskal–Wallis test revealed a non-significant difference of the number of urine EV by NTA among all groups. **b** Size distribution of EV isolated from the urine, Kruskal–Wallis test revealed a non-significant difference in the size of urine EV among all groups. **c** Average peak concentration over the size of extracted EV. **d** Transmission-Electron Microscopy (TEM) micrograph of a negatively stained representative EV sample. Arrows indicate EV.

NTA showed comparable particle concentrations in all four study groups (AR, CR, ST, and healthy controls), indicating that subsequent proteomic differences were not attributable to variation in EV abundance (Fig. 1A). The vesicles displayed a narrow size distribution centered at ~200 nm (modal diameter198 ± 21 nm), with no statistically significant between-group differences (Fig. 1B). An Average peak concentration over the size of extracted EV is demonstrated in Fig. 1C. Transmission-electron microscopy corroborated these findings, revealing the expected cup-shaped morphology and confirming the NTA-derived size estimates (Fig. 1D). Label-free LC-MS/MS identified 3196 unique proteins; 82–89% overlapped with entries in established EV databases (EVpedia, Vesiclepedia, ExoCarta). In addition to generic exosomal markers(CD9, CD63, CD81- see supplementary fig), the dataset contained kidney-specific transporters and channels, including aquaporin-1, aquaporin-2, ATP6V0D2, and the betaine/GABA transporter SLC6A12, consistent with the renal origin of the vesicles.

### Differential proteomic profile of urinary EVs

Urinary EVs from AR patients demonstrated a unique proteomic profile characterized by predominantly upregulated proteins compared to other groups. The number of differentially expressed proteins for each pairwise group comparison is summarized in Table 2.

**Table 2:** Number of differentially expressed proteins in each pairwise comparison among the patient groups

Comparison	Up	Down	Any
**ST vs. Control**	0	0	0
**AR vs. Control**	63	4	67
**CR vs. Control**	2	0	2
**AR vs. ST**	150	27	177
**CR vs. ST**	0	0	0
**AR vs. CR**	10	1	11
**Any comparison**			192

Proteins were considered differentially expressed if they had FDR-adjusted *p* value < 0.05.

**Fig. 2:**
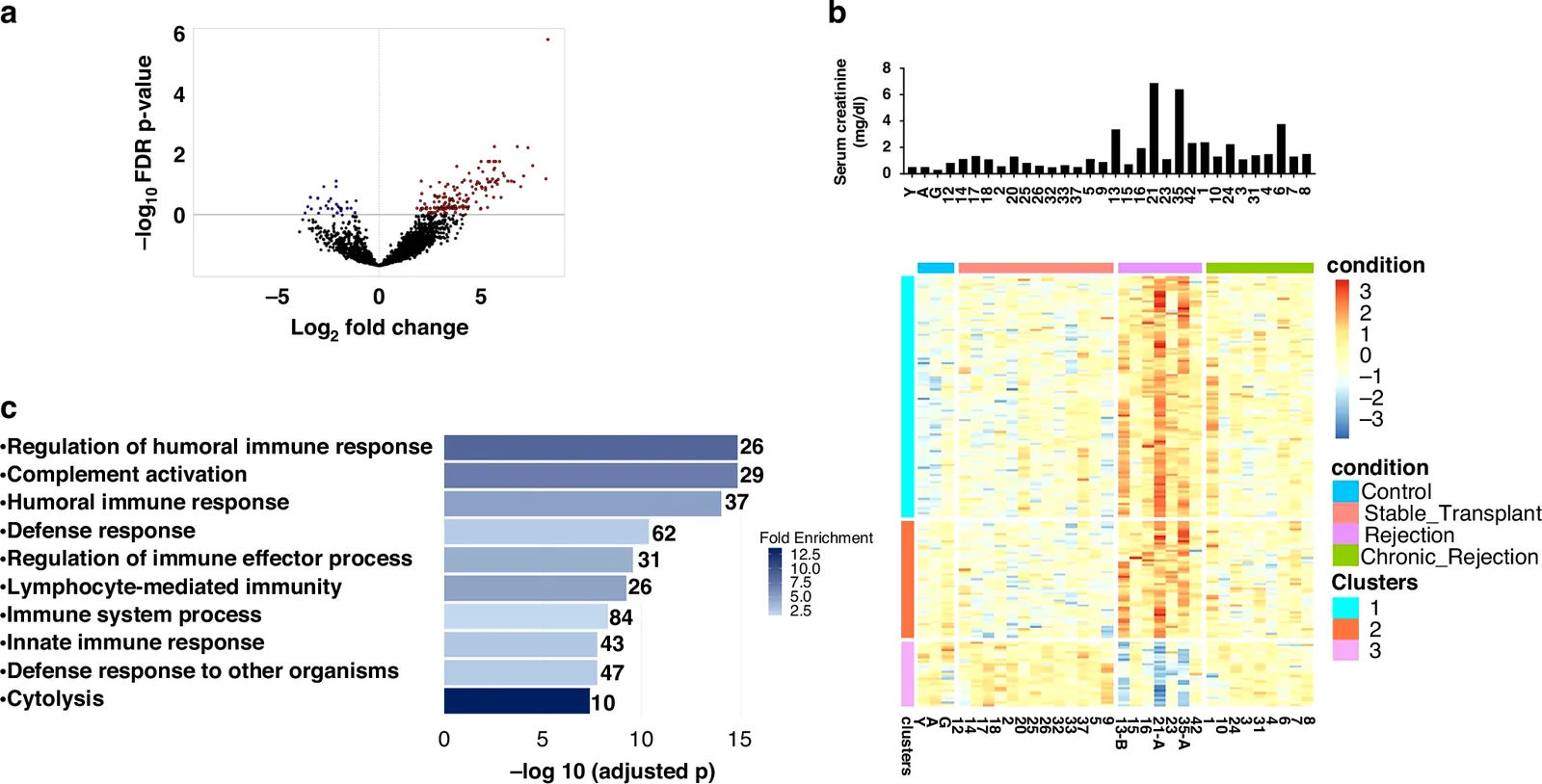
LC/MS proteomics profiling of urinary EV. **a** Volcano plot showing log_2_ fold change and FDR-adjusted *p* values of the comparison AR vs. ST. Up- and down-regulated proteins (FDR-adjusted *p* value < 0.05, depicted above a horizontal cutoff line) are marked in red. **b** Clustering analysis applied to differentially expressed proteins between AR and ST, revealing three distinct clusters. Each column represents a single patient, and each row represents a specific protein. The upper part depicts the serum creatinine levels. **c** Top 10 results of GO BP enrichment analysis of AR vs. ST upregulated proteins sorted by adjusted *p* values. The number of upregulated proteins for each GO term is indicated next to the bars. Bars are shaded according to fold enrichment. No significant GO BP enrichment was found among the down-regulated proteins.

The AR vs. ST comparison yielded the largest number of differentially expressed proteins, identifying 177 in total. Their fold-change and *p* value distribution is shown in the volcano plot (Fig. 2A). Hierarchical clustering of this protein set grouped them into three major clusters (Fig. 2B). Clusters A and B comprised of upregulated proteins in AR, whereas cluster C contained downregulated proteins in AR.

Notably, the two upregulated clusters exhibited distinct patterns within the ST group: proteins in cluster A remained above control levels, while those in cluster B declined below control levels, suggesting divergent biological processes within the overall AR response. Importantly, the AR-specific protein signature showed a strong correlation with changes in blood creatinine levels (Fig. 2B), highlighting its potential diagnostic relevance. The CR group demonstrated a proteomic profile more consistent with the stable group vs AR group. (Fig. 2B).

### Pathway enrichment

GO over-representation analysis identified 125 biological processes enriched among AR-elevated proteins (FDR < 0.05; minimum count = 5). Dominant categories included humoral-immune response, complement activation, classical pathway activation, antigen processing and presentation, and cellular stress responses (Fig. 2C; Supplementary Table S1). Together, these findings delineate a robust, immune-centered urinary EV proteomic pattern that differentiates AR from both CR and stable graft function. These findings are consistent with known mechanisms of alloimmune injury and provide functional context for the protein-level alterations observed.

### Longitudinal dynamics of the EV proteome

**Fig. 3:**
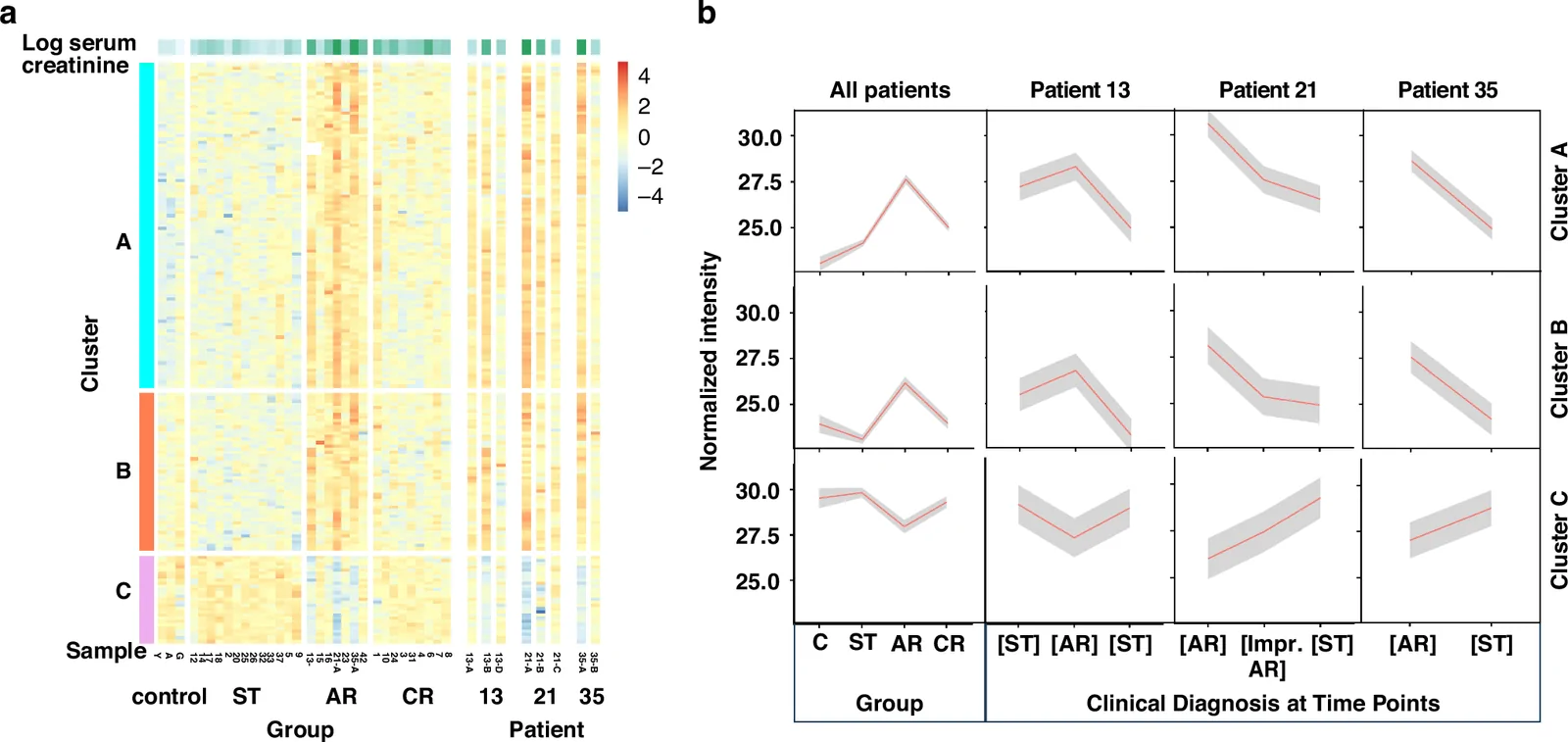
Proteomic profiles of longitudinal samples from individual patients. **a** A heatmap representing the proteome profile of repeated samples from the 3 patients (# 13, 21, 35), shown next to the clustering heatmap of all patients in the study by patient group. **b** Average expression profile per cluster. Left: for all patients, across patient groups. Right: per patient, along time points. Note that in both (**a**, **b**), the clustering was calculated using “all patients”, such that from each of the patients having repeated samples only one sample (13B, 21 A and 35 A) was considered.

Serial sampling in three recipients allowed us to examine how the EV proteome evolves before, during, and after a rejection episode (Fig. 3).

#### Case 1

-Patient 13. This 14-year-old boy was evaluated for rejection three weeks after his second living-donor transplant for recurrent focal-segmental glomerulosclerosis (FSGS). At enrollment, he had normal renal indices (Creatinine0.7 mg/dl; sample13 A, Fig. 3A, B) and no proteinuria. The urinary EV profile, however, already resembled the AR signature identified in cross-sectional analyses. Three weeks later, his creatinine rose sharply to 4.0 mg dl, and biopsy-confirmed Banff grade 2B acute cellular rejection (sample 13B). High-dose steroids plus anti-thymocyte globulin produced clinical improvement, with creatinine falling to 1.2 mg dl. Four months later (sample 13 C), the EV proteome had reverted to the stable-graft pattern, mirroring his biochemical recovery.

#### Case 2

-Patient 21. A 15-year-old girl received a maternal graft for congenital renal hypodysplasia. Acute rejection occurred on day 5 post-transplant: creatinine climbed from 1.3 to 5.4 mg dl and biopsy showed Banff 2 A rejection. Both the index sample (21 A) and a repeat taken five days later (21B; creatinine 4.2 mg dl) displayed the characteristic AR proteome. Three months after successful treatment (creatinine 1.1 mg dl, her third sample (21 C) clustered with the stable-function group, indicating molecular resolution of the rejection process.

#### Case 3

-Patient 35. This 16-year-old boy was enrolled eight days after a deceased-donor transplant for renal dysplasia. An early biopsy, performed for rising creatinine (1.4 → 3.5 mg dl), revealed Banff2 A rejection with superimposed acute tubular necrosis (sample35 A). Pulse steroids and thymoglobulin were followed by clinical improvement, and six months later, a protocol biopsy was normal (creatinine1.4 mg dl). The EV proteome in the convalescent sample (35B) aligned with the stable-graft reference set.

Across all three cases, the AR-specific EV signature was detectable before or contemporaneous with the earliest biochemical changes and disappeared once histological and functional recovery had occurred. These observations support the utility of urinary EV profiling for real-time, non-invasive monitoring of graft integrity and suggest that proteomic shifts may precede conventional markers by several weeks.

## Discussion

Children who receive a kidney transplant confront a markedly higher incidence of AR than adults and, because of their longer life expectancy, will almost certainly need more than one graft during their lifetime.^2,24^ Although protocol biopsies remain the diagnostic gold standard, their invasive nature, the requirement for general anesthesia, and non-negligible bleeding risk make them problematic in pediatric practice.^25,26^ In this context, our study offers the first comprehensive characterization of the urinary EV proteome across ST function, CR, and AR in children.

We identified 3196 unique EV proteins, 82–89% of which overlap with established EV databases, demonstrated a 177-protein signature that separates AR from ST, and showed that the same signature rises weeks before histological rejection and normalizes after therapy. These findings provide a compelling rationale for deploying urinary EVs as a non-invasive, organ-proximal biomarker platform.

NTA revealed a trend toward higher EV counts in AR, echoing the adult observations of Burrelloet al.^27^. However, the difference did not reach statistical significance, consistent with the report by Sedej et al.^28^. Taken together with our data, these studies indicate that vesicle quantity alone is unlikely to provide diagnostic discrimination and that molecular cargo must be interrogated to realize the full biomarker potential of EVs.

The AR-associated proteins were strongly enriched for humoral-immune and complement pathways, particularly classical pathway activation and interferon-γ signaling, corroborating the transcriptomic findings of Limet al.^29^ and matching the histological footprint of TCMR. At the same time, aquaporin-2, SLC12A1, and other tubular transporters were depleted, reflecting epithelial stress that accompanies immune injury. The convergence of pathway analysis with established rejection biology supports the mechanistic validity of the EV signal.

Our results agree with adult EV studies: Sigdel et al. identified 11 urinary EV proteins, three of which were exclusive to the vesicle fraction, that discriminated biopsy-proven AR ^15^; Fekihet al. described a proteomic shift in urinary EV during allograft rejection,^30^ and Park et al. highlighted complement and HLA enrichment in adult TCMR.^31^ Beyond EVs, donor-derived cell-free DNA (dd-cfDNA) assays achieve area-under-the-curve (AUC) values of 0.74–0.87 in adults but requires venipuncture and high-cost sequencing.^32^ Urinary chemokines such as CXCL10 reach AUCs of 0.78–0.82 yet lose specificity in the presence of infection or delayed graft function.^32^

Serial sampling in three children showed that the EV profile shifted toward the AR pattern a median of 18 days before creatinine rose or biopsy was performed and returned to the ST pattern after histological resolution. Similar temporal behavior has been hinted at in adult cohorts ^28,31,33^ but has never before been demonstrated in pediatric recipients.

We acknowledge the limitations of this study, which are inherent to its design as a single‑center pilot. These include the modest sample size, the potential for selection bias, and the exclusive use of ultracentrifugation - a method that may underrepresent EVs smaller than 100 nm. Furthermore, the study was not powered to analyze ABMR in detail or to assess the impact of intercurrent infections. Given our center doesn’t perform surveillance biopsies, we may have missed early rejection findings in the stable allograft group. Validation in larger, multicenter cohorts will be essential before clinical application can be contemplated.

Notwithstanding these constraints, the study offers notable strengths. We achieved extensive, label‑free proteomic profiling ( > 3100 proteins) with rigorous FDR control, and we incorporated serial sampling, enabling us to track dynamic molecular trajectories over time.

If corroborated in larger studies, EV monitoring holds several potential clinical benefits: (i) reducing the need for invasive biopsies by reliably identifying periods of low risk; (ii) enabling pre‑emptive intervention when molecular signatures indicate rising risk; and (iii) providing an objective metric for evaluating treatment response.

### Future directions

The diagnostic performance of the urinary‑EV signature needs to be verified in prospective cohorts drawn from multiple transplant centers and geographic regions. Such studies should be powered to generate precise estimates of sensitivity, specificity, and predictive values across diverse immunological‑risk strata, age groups, and ethnic backgrounds. Harmonized inclusion criteria and central pathology review will be essential to ensure comparability of results.

Beyond TCMR, urinary EVs may harbor distinct proteomic patterns for ABMR, BK-virus nephropathy, and calcineurin-inhibitor toxicity. Targeted sub‑analyses, ideally with biopsy‑proven phenotypes and virological data, will determine whether tailored panels can improve differential diagnosis and guide phenotype‑specific therapy.

For urinary‑EV diagnostics to reach routine clinical use, reproducibility across laboratories must be demonstrated. This will require consensus on pre‑analytical variables (sample timing, storage, and handling), EV isolation and enrichment methods, protein‑quantification platforms, and data‑normalization strategies. International working groups should develop reference materials and external‑quality‑assessment schemes to support regulatory approval and widespread implementation.

## Conclusions

Urinary EV proteomics captures the molecular footprint of AR in PKT, anticipates functional decline, and mirrors histological recovery. By offering a sensitive, organ-specific, and truly non-invasive monitoring tool, EV-based assays could transform pediatric graft surveillance and improve long-term graft survival. Rigorous multicenter validation is now warranted to translate these promising preliminary findings into clinical practice.

## Supplementary information

Supplementary.png.pdf

Supplemental Table

## Data Availability

The dataset generated and analyzed during the current study is available from the corresponding author on reasonable request.
