# Supplementary material for: Detecting hidden threats: proteomic changes in urinary extracellular vesicles are associated with pediatric kidney allograft rejection
Source: Pediatr Res. 2026 Apr 22;100(2):856–62. doi: 10.1038/s41390-026-04866-z (PMC13638442; doi:10.1038/s41390-026-04866-z)
Supplement: Supplementary file 1 [file 41390_2026_4866_MOESM1_ESM.pdf]

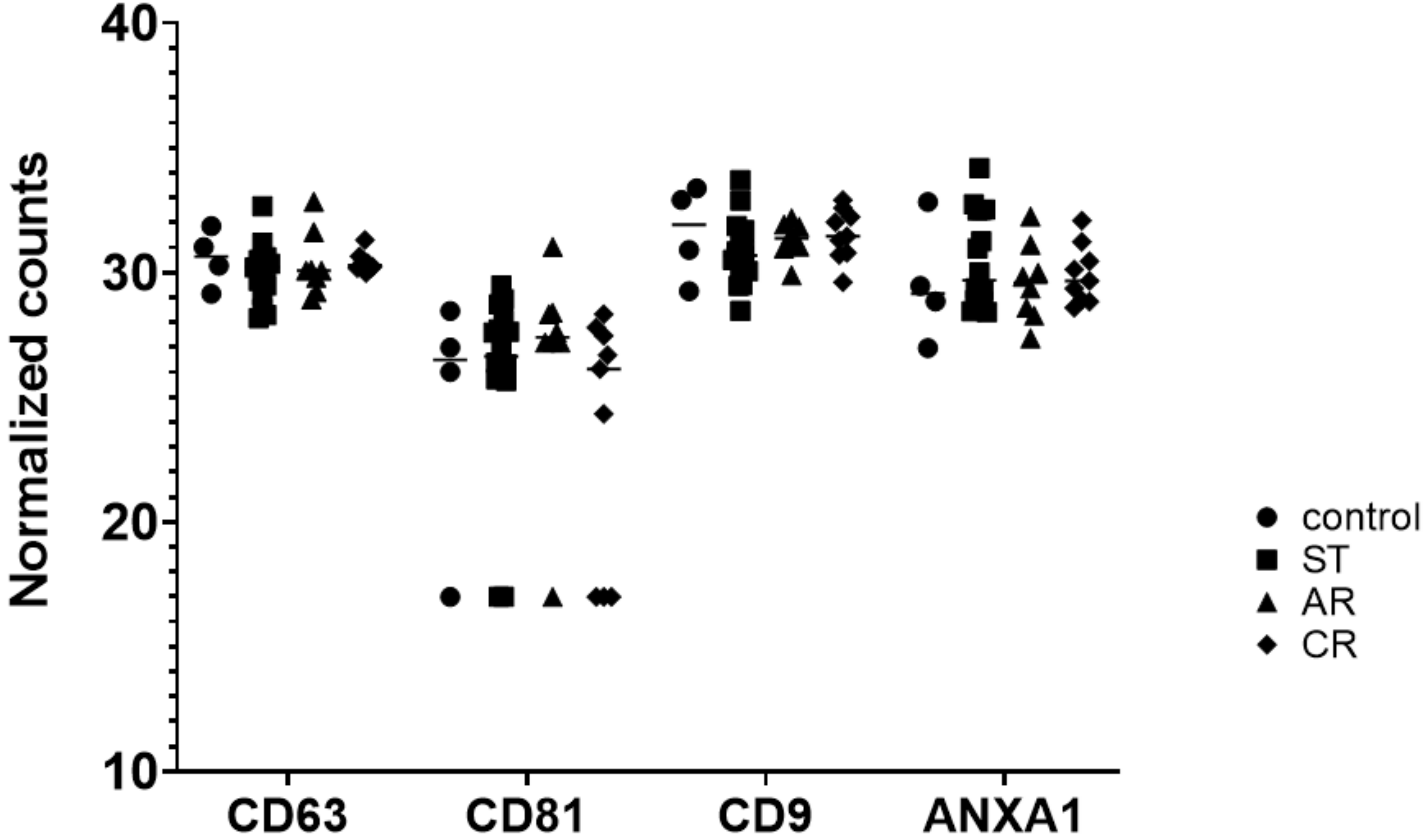

Normalized counts
40
30
20
10
CD63
CD81
CD9
ANXA1
control
ST
AR
CR
